# Immunoproteomic to Identify Antigens in the Intestinal Mucosa of Crohn's Disease Patients

**DOI:** 10.1371/journal.pone.0081662

**Published:** 2013-12-16

**Authors:** Zheng Zhou, Haiyan Liu, Guosheng Gu, Gefei Wang, Wenyong Wu, Changle Zhang, Jianan Ren

**Affiliations:** 1 Department of General Surgery, First Affiliated Hospital of Anhui Medical University, Anhui, Hefei, P.R. China; 2 Department of Critical Care, First Affiliated Hospital of Anhui Medical University, Anhui, Hefei, P.R. China; 3 Research Institute of General Surgery, Jinling Hospital, Medical School of Nanjing University, Nanjing, P.R. China; Queensland Institute of Medical Research, Australia

## Abstract

Incidences of Crohn disease (CD) have increased significantly in the last decade. Immunoproteomics are a promising method to identify biomarkers of different diseases. In the present study, we used immunoproteomics to study proteins of intestinal mucosal lesions and neighboring normal intestinal mucosa of 8 CD patients. Reactive proteins were validated by Western blotting. Approximately 50 protein spots localized in the 4 to 7 pI range were detected on two-dimensional electrophoresis gels, and 6 differentially expressed protein spots between 10 and 100 kDa were identified. Reactive proteins were identified as prohibitin, calreticulin, apolipoprotein A-I, intelectin-1, protein disulfide isomerase, and glutathione s-transferase Pi. Western blotting was conducted on the intestinal mucosa of another 4 CD patients to validate the reactive proteins. We found that intestinal mucosal lesions had high levels of prohibitin expression. Glutathione s-transferase expression was detected in 100% of the intestinal mucosa examined. Thus, we report 6 autoantigens of CD, including 3 new and 3 previously reported autoantigens. Intelectin-1, protein disulfide isomerase, and glutathione-s-transferases may be used as biomarkers for CD pathogenesis.

## Introduction

Crohn disease (CD) is a relapsing inflammatory disease that mainly affects the gastrointestinal tract. CD frequently presents with abdominal pain and fever, and it sometimes may lead to life-threatening complications. Genetic, environmental, and immunological factors play important roles in CD development. CD affects about 500,000 persons in North America [1]. In Asia, CD prevalence ranges from 3.6 to 7.7 per 100,000 people [2], [3]. In a European multinational population-based study, Wolters et al. revealed an increased mortality risk in CD patients 10 years after diagnosis. Age over 40 years at diagnosis was the sole factor associated with increased mortality risk [4]. Although cellular and biological methods have greatly improved our understanding of the pathogenesis of CD [5]–[8], no single distinct mechanism can explain all aspects of this disease. Given the increase in CD cases worldwide, more accurate biomarkers for diagnosis are needed to improve the efficiency of diagnosis and enable early treatment. Immunoproteomics, a technique involving two-dimensional electrophoresis (2-DE) followed by immunoblotting, holds considerable promise for the discovery of biomarkers in different diseases, including cancer, autoimmune diseases, and infections [9], [10]. This technique has been used to identify immune-associated antigens, as well as their isoforms and posttranslational modifications. Recently, the immunoproteomic approach has been used to identify autoantibodies for neuropsychiatric systemic lupus erythematosus, Hashimoto's encephalitis and multiple sclerosis [11]–[13]. In this study, we applied an immunoproteomic approach to survey proteins of the intestinal mucosa in CD patients and identified 6 autoantigens.

## Materials and Methods

### Clinical specimens

#### Ethical statement

Study protocols were approved by the medical ethics committee of Jinling Hospital, Medical School of Nanjing University. Written informed consent was obtained from all subjects prior to participation. Clinical serum specimens were obtained from 8 CD patients (mean CDAI[Crohns Disease Activity Index] score: 344) at the time of diagnosis. Specimens were processed promptly after collection and stored at −70°C until analysis. Intestinal mucosal lesions were obtained via surgery; neighboring normal intestinal mucosal tissue served as the control group.

#### Preparation of intestinal mucosa

Intestinal mucosa was scraped with a glass microscope slide and stored at −80°C until just prior to starting 2-D polyacrylamide gel electrophoresis (PAGE). For total protein extract preparation, mucosal scrapings were homogenized in solubilization buffer at 4°C (1% Triton X-100, 100 mM Tris-HCl [pH 7.4], 100 mM sodium pyrophosphate, 100 mM sodium fluoride, 10 mM EDTA, 10 mM sodium orthovanadate, 2.0 mM phenylmethylsulfonyl fluoride [PMSF], and 0.1 mg aprotinin/mL) with a Polytron PTA 20S generator (model PT 10/35; Brinkmann Instruments, Westbury, NY) operated at maximum speed for 30 seconds. The material was centrifuged for 20 min at 110,000 rpm at 4°C). The protein concentration in supernatants was determined by the Bradford method.

### Protein separation by 2-D PAGE

Intestinal mucosal proteins (60 µg) were diluted with rehydration solution (8 M urea, 2% CHAPS, 65 mM DTT, 0.5% vol/vol isoelectric focusing [IEF] buffer [pH 4–7], trace bromophenol blue) to a final volume of 250 ml. IEF was performed using a Protean IEF system (Amersham Bioscience, Sweden). Gels were rehydrated at 30 V for 6 hours and at 60 V for 6 hours. Proteins were subsequently focused for 1 hour at 500 V and 1 hour at 1000 V; then, a gradient was applied from 1000 to 8000 V for 1 hour; finally, the voltage was set at 8000 V to subject the samples to a total of 30,000 V h. All IEF experiments were performed at 20°C.

After one-dimensional IEF, IPG strips were placed in an equilibration solution (6 M urea, 2% sodium dodecyl sulfate [SDS], 30% glycerol, 1.5 M Tris-HCl, pH 8.8) containing 2% DTT and were shaken for 15 minutes at 50 rpm. The strips were transferred to an equilibration solution containing 2.5% iodoacetamide, shaken for 15 minutes, transferred to vertical slabs of 12.5% SDS-PAGE gels, and sealed with 0.5% low-melting-point agarose. After electrophoresis, proteins in gels were transferred to a PVDF membrane and the rest of proteins in gels were visualized by silver staining. Images were digitized using a GS-800 Calibrated Densitometer (Bio-Rad, USA) and analyzed with the PDQuest 2-D Analysis software package. To minimize variation in sample loading and staining, 2-DE analysis was repeated 3 times.

### Detection of CD antigens by Western blotting

After protein transfer, the PVDF membranes were incubated for 2 hours in blocking buffer comprising 5% milk in 10 mM Tris-HCl (pH 7.5), 2.5 mM EDTA (pH 8.0), and 50 mM NaCl. Membranes were incubated with serum obtained from CD patients as the source of primary antibodies at 1∶200 dilution under room temperature (RT) for 2 hours. Serum samples from all 8 patients were mixed together. After 3 washes with washing buffer (Tris-buffered saline containing 0.01% Tween 20), membranes were incubated with horseradish peroxidase (HP)-conjugated antihuman IgG antibodies (Beijing Zhongshan Company, China) at a dilution of 1∶5,000 for 1 hour at RT. Proteins in intestinal mucosal lesions were compared directly with proteins from the neighboring normal intestinal mucosa.

### In-gel enzyme digestion and mass spectrometry (MS)

Each differentially expressed spot from the gels was dehydrated with 50 ml of ACN for 5 min, incubated in 50 mL of 10 mM DTT at 56°C for 1 hour, and then incubated in 50 mL of 55 mM iodoacetamide (alkylating solution) for 45 min. The spots were dehydrated with 50 ml of ACN and rehydrated in 5 ml of porcine trypsin, followed by the addition of 10 ml of 25 mM ammonium bicarbonate. Proteolysis was performed overnight at 37°C and stopped by adding 10 ml of 2% formic acid. Resulting peptides were concentrated and mixed with alpha-cyano-4-hydroxycinammic acid (Sigma, St. Louis, MO, USA), deposited on a 384-well MALDI target, and air-dried. Analyses were performed with a Biflex IV (Bruker Daltonics, Germany). MS data were compared against tryptic peptide sequences from the SWISS-PROT database using Mascot (Matrix Sciences, London, UK) search algorithms.

### Western blot analysis

Each serum sample was loaded at the same concentration. Serum proteins were subjected to 12.5% SDS-PAGE and then transferred onto polyvinylidene difluoride (Millipore, Billerica, MA) membranes, which were blocked for 30 minutes with 5% nonfat milk in Tris-buffered saline with 0.05% Tween-20 (TBST). Samples were incubated overnight at 4°C in 1∶400 v/v solutions of mouse antihuman prohibitin (Abcam UK) or mouse antihuman glutathione S-transferase (GST) Pi (Abcam UK). The membranes were washed 3 times in TBST for 5 minutes each and blotted for 2 hours with a 1∶500 solution of HP-conjugated secondary rabbit antimouse IgG at RT. The membranes were washed twice with TBST, followed by another wash in TBS. Immunoreactive bands were visualized with a Western Blotting Detection System (Pierce, Rockford, IL). Protein mass was compared after quantifying the intensity of protein bands by Quantity One software (Bio-Rad).

## Results

### Autoantibodies to intestinal mucosa proteins

**Figure 1:**
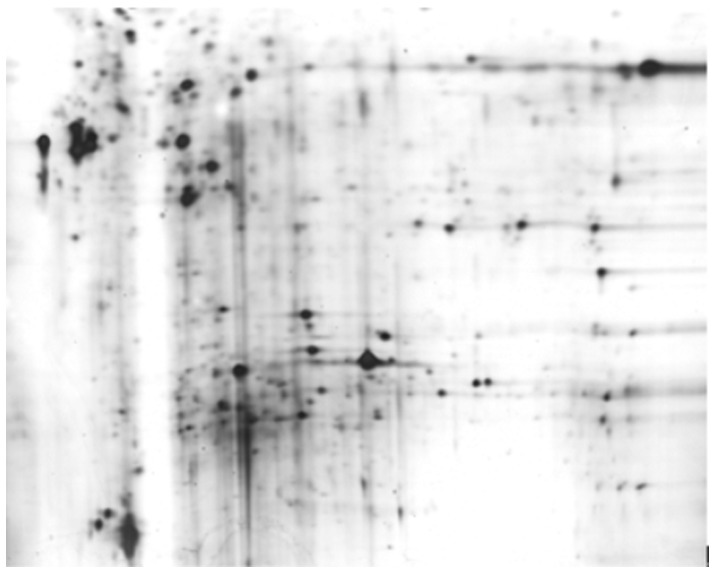
2-DE analysis of proteins from intestinal mucosal lesions in patients with CD.

Total protein extracts of intestinal mucosal lesions and neighboring normal intestinal mucosa, obtained from 8 CD patients with a mean CADI score of 344, were separated by 2-D PAGE and transferred to PVDF membranes. Serum samples from 8 CD patients were mixed. The serum proteins were the primary antibody and rabbit antimouse IgG was the secondary antibody in our assay. Approximately 50 protein spots localized in the 4–7 pI range were detected on the 2-DE gels. Six differentially expressed protein spots between 10 and 100 kDa were identified (Fig. 1).

### Identification of the reactive proteins

**Figure 2:**
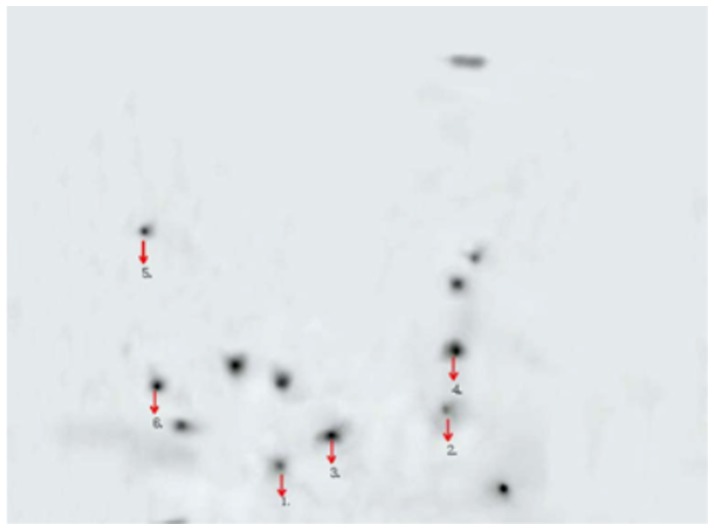
Detection of intestinal mucosal antigens by Western blot analysis in CD patients. The six protein spots are labeled with arrows.

Proteins of interest were extracted from the gels after 2-D PAGE and silver staining. The proteins were digested with trypsin, chymotrypsin, and Glu-C, and the resulting peptides were analyzed by MALDI-TOF MS. The corresponding spectra were used to identify the proteins with the Mascot search program. The reactive proteins were identified as prohibitin, calreticulin, apolipoprotein A-I, intelectin-1, protein disulfide isomerase (PDI), and GST Pi (Fig. 2, Table 1).

**Table 1:** Differentially Expressed Proteins Identified by MALDI-TOF-MS.

accession number	Sequence coverage	Score	p*I*/*Mr*	Protein name
NP_000843	50%	81	5.43/23569	Glutathione S-transferase Pi
AAS88903	76%	237	5.57/29859	Prohibitin
NP_000030	64%	169	5.56/30759	Apolipoprotein A-I precursor
NP_060095	42%	89	5.66/35510	Intelectin-1 precursor
NP_004334	47%	165	4.29/48283	Calreticulin precursor
NP_000909	32%	116	4.76/57480	Protein disulfide-isomerase precursor

### Western blot analysis to validate the reactive proteins

**Figure 3:**
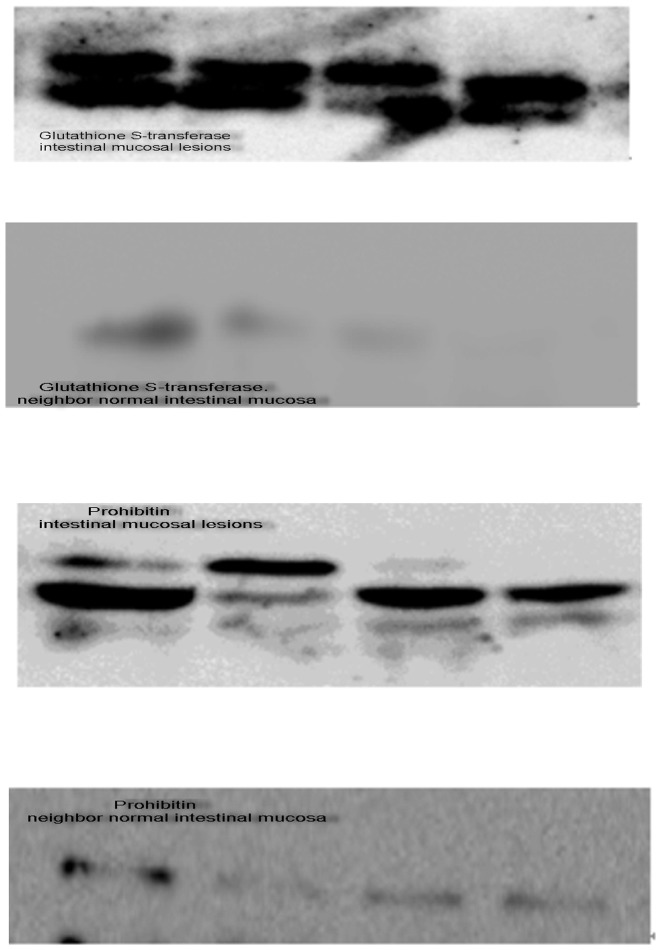
Western blot analysis to validate the reactive proteins. Intestinal mucosal lesions and neighboring normal intestinal mucosa were examined by Western blot analysis to validate the reactive proteins. Samples were probed for (A) prohibitin or (B) GST. Note the higher expression levels of prohibitin and GST in intestinal mucosal lesions. Multiple bands in Western blot analysis were correlated with protein degradation or modification.

To confirm the immunoproteomic data, intestinal mucosal lesions from another 4 CD patients (mean CADI score = 244) were analyzed by Western blot. Intestinal mucosal lesions had high levels of prohibitin expression (Fig. 3A). GST expression was detected in 100% (4/4) of the intestinal mucosa examined (Fig. 3B). Multiple bands were observed in samples of intestinal mucosal lesions, indicating protein modification or degradation. Comparison of intestinal mucosa from the two groups demonstrated that both prohibitin and GST were present in intestinal mucosal lesions from CD patients.

## Discussion

Identification of disease-specific autoantigens is essential for understanding the pathogenesis of autoimmune diseases and defining biomarkers for detection of preclinical autoimmune disorders. With the rising incidence of CD, novel biomarkers are required for better diagnosis of CD. In this study, immunoproteomics revealed 6 autoantigens for CD in the intestinal mucosal lesions of CD patients. In particular, human intelectin-1, PDI(Protein Disulfide Isomerase), and GST(Glutathione S-transferase Pi) have not previously been reported as CD autoantigens. These proteins may be used as biomarkers for CD pathogenesis. Our immunoproteomics approach for autoantibody profiling of autoimmune diseases is a convenient and effective tool for detecting autoantigens.

Three antigenic proteins, prohibitin, calreticulin and apolipoprotein A-I, have already been reported in CD. Prohibitin (PHB) is a member of the Band-7 family of proteins; it is highly conserved evolutionarily, widely expressed, and present in different cellular compartments. PHB plays a pivotal role in the processes of cell differentiation and apoptosis [14], [15]. Theiss et al. found that PHB primarily localizes to the mitochondria in intestinal epithelial cells, constituting a cellular defense against oxidant injury [16]. In another study investigating the potential role of PHB in modulating mitochondrial stress-related autophagy in intestinal epithelial cells, Kathiria et al. found that decreased PHB levels coupled with dysfunctional autophagy rendered intestinal epithelial cells susceptible to mitochondrial damage and cytotoxicity. They determined that repletion of PHB may represent a therapeutic approach to combat oxidant and cytokine-induced mitochondrial damage in inflammatory bowel disease [17].

Calreticulin (CRT) is a soluble Ca^2+^-binding protein present in various cellular signaling pathways [18]. CRT is localized mainly in the endoplasmic reticulum (ER) and is often a target for autoantibodies in patients with autoimmune disease. In a study to evaluate the clinical significance of anti-CRT antibodies using the serum of patients with IBD, Watanabe et al. found the mean titer of anti-CRT antibodies was higher in patients with CD than in healthy individuals. They presumed that the positivity of anti-CRT antibodies may have a diagnostic value for IBD [19].

Apolipoprotein A-I is the major protein component of high-density lipoprotein particles and the primary acceptor for cholesterol in extrahepatic tissues. Levy et al. examined the lipoprotein composition in 22 pediatric CD patients and 10 healthy control subjects. Plasma apolipoproteins A-I levels were lower in CD patients, and lipoprotein concentrations were altered in CD patients [20]. In other studies, serum apolipoprotein A-I was upregulated compared to baseline samples in CD patients when the patients were treated with infliximab [21], [22].

Human intelectin-1 (ITLN-1) is a novel identified galactose-binding lectin that is usually expressed in the heart, small intestine, and colon [23]. In normal colon epithelia, human ITLN-1 is produced from the goblet cells and secreted into mucus [24], and the ITLN-1 expression increases during gastrointestinal infection [25], [26]. Recombinant ITLN-1 possesses an affinity to D-pentose and recognizes the bacterial arabinogalactan of Nocardia containing D-galactofuranosyl residues [23]. Previous studies indicated that both human and mouse intelectin-1 may play immunological roles in the defense against selected microorganisms or foreign antigens [25], [27], [28]. More importantly, recent evidence showed overexpression of ITLN-1 in human malignant pleural mesothelioma, which has potential screening applications in differentiating this disease from lung cancer [24], [29]. Because gastric adenocarcinomas can arise through an intestinalization process [30], and considering the evidence that ITLN-1 is expressed in colonic goblet cells [24], we hypothesize that ITLN-1 may be aberrantly expressed in human gastric cancer and involved in the carcinogenic events associated with gastrointestinal malignancies.

PDI is a multifunctional enzyme that primarily catalyzes disulfide bond formation, breakage, and rearrangement [31]. Disulfide bonds stabilize the structure of proteins and can regulate the activity of various enzymes [32]. PDI is primarily associated with the ER, where it participates in protein folding during biosynthesis. It is also found on the cell membrane and may be actively secreted by various cell types [32], [33]. Extracellular PDI has been shown to regulate numerous activities, including cellular adhesion [34], pathogen entry [35], [36], platelet aggregation and secretion [37], intracellular nitric oxide delivery [38], and insulin degradation [39], [40]. Lymphocytes (especially CD4+ T cells) increase the availability of cell surface thiols after immune activation [41]. An intriguing study recently showed that galectin-9 binding to PDI on Th2 cells induced increased cell migration through the extracellular matrix via β3 integrins, identifying a unique mechanism to regulate T-cell migration [42].

GSTs comprise a family of phase II enzymes that can conjugate glutathione hormone (GSH) with various environmental etiological factors. GST is ubiquitously expressed in normal and malignant tissues [43]. The tissue- and species-specific expression and distribution of GSTs are an adaptive response against the toxicity of endogenous and exogenous metabolites [44]. In a molecular biology experiment, intestinal epithelial Caco-2 cells were cocultured with lipopolysaccharide from Escherichia coli (EC-LPS). EC-LPS challenge induced a decrease in GST-alpha (GSTA1/A2) mRNA levels, protein expression, and catalytic activity; butyrate treatment restored GSTA1/A2 mRNA levels, protein expression, and catalytic activity [45]. In an epidemiological investigation aimed to evaluate GSTM1 and GSTT1 in patients with IBD and healthy controls from northern India, Mittal et al. found that null genotypes of GSTM1 and T1 were associated with IBD. The combination of the two GST genotypes further increased the risk [46]. However, in a study examining associations between the detoxifying GST family mu, theta, and pi gene variants and IBD, the GST genotypes were not important in susceptibility to IBD in a Danish population [47].

However, the limitations of the present study should be acknowledged. First, the number of CD patients was too small to generalize current findings to a large population. As a result, all results need to be confirmed in further study with large sample size. Second, the lack of controlled patients and follow up data makes it difficult to choose more accurate autoantigens. In conclusion, our proteomic analysis of intestinal mucosa in CD identified 6 autoantigens, including 3 new autoantigens as well as 3 previously reported autoantigens. We suggest the 3 new autoantigens: human intelectin-1, PDI and GST may be used as biomarkers for CD pathogenesis.
